# Food Store Environment Modifies Intervention Effect on Fruit and Vegetable Intake among Low-Income Women in North Carolina

**DOI:** 10.1155/2012/932653

**Published:** 2012-01-17

**Authors:** Alison A. Gustafson, Joseph Sharkey, Carmen D. Samuel-Hodge, Jessica C. Jones-Smith, Jianwen Cai, Alice S. Ammerman

**Affiliations:** ^1^Department of Nutrition and Food Science, College of Agriculture, University of Kentucky, 206G Funkhouser, Lexington, KY 40506, USA; ^2^Program for Research in Nutrition and Health Disparities, School of Rural Public Health, Texas A&M Health Science Center, College Station, TX, USA; ^3^The Department of Nutrition, Gillings School of Global Public Health and Center for Health Promotion and Disease Prevention, University of North Carolina, Chapel Hill, NC 27599, USA; ^4^School of Medicine and Center for Health and Promotion, University of California San Francisco, San Francisco, CA 94143, USA; ^5^Department of Biostatistics, Gillings School of Global Public Health, University of North Carolina at Chapel Hill, Chapel Hill, NC 27599, USA

## Abstract

*Background*. The aim of the study is to determine how the food store environment modifies the effects of an intervention on diet among low-income women. *Study Design*. A 16-week face-to-face behavioral weight loss intervention was delivered among low income midlife women. *Methods*. The retail food environment for all women was characterized by (1) the number and type of food stores within census tracts; (2) availability of healthy foods in stores where participants shop; (3) an aggregate score of self-reported availability of healthy foods in neighborhood and food stores. *Statistical Analyses*. Multivariable linear regression was used to model the food store environment as an effect modifier between the intervention effect of fruit and vegetable serving change. *Results*. Among intervention participants with a low perception of availability of healthy foods in stores, the intervention effect on fruit and vegetable serving change was greater [1.89, 95% CI (0.48, 3.31)] compared to controls. Among intervention participants residing in neighborhoods with few super markets, the intervention effect on fruit and vegetable serving change was greater [1.62, 95% CI (1.27, 1.96)] compared to controls. *Conclusion*. Results point to how the food store environment may modify the success of an intervention on diet change among low-income women.

## 1. Introduction

Strategies and methods aimed at improving fruit and vegetable intake have begun to consider the environment as a potential level for change [1]. It has been suggested that studies are needed for measuring the interaction between the individual and their environment to disentangle the effects of environmental characteristics from individual behaviors [2]. Cogent to reviews suggesting where the field needs to move [2–4], there has been a large upswing in environmental level interventions as a method for understanding and improving outcomes for weight loss and improving diet quality [3]. Yet, to date there is little research examining how the food store environment may interact or modify the relationship between individual level interventions with diet and weight outcomes [5].

Several studies thus far have addressed how individual level variables such as cooking skills [6], meal planning [7, 8], and social support [9] may influence or modify the effect of a behavioral dietary change intervention. Results have found that meal planning may modify the success of dietary change [7, 8] and that social support is a strong factor in purchasing of fruits and vegetables [9] as well as consumption [10]. Research to date has examined socioeconomic status modifies the relationship between the food environment and weight [11]. However, there are no published studies examining how the food store environment may be a modifier between a behavioral weight and diet change intervention with diet change.

Fruit and vegetable consumption has been shown to vary across neighborhoods suggesting that beyond individual factors as discussed above, environment level factors such as access to stores selling fruits and vegetables may play a role in consumption. Additionally, several studies have found that low-income individuals and rural populations are disproportionally affected by poor access and availability of fruits and vegetables [12–14]. It is suggested that the lack of access to healthy food options prohibits individuals from obtaining and consuming nutrient dense items, such as fruits and vegetables [15]. However, given the lack of research examining how environmental factors may modify the effect of dietary change interventions among low-income populations, there is a gap in understanding the interaction between the individual and their environment.

The aim of this study is to determine how perceived and objective measures of the food store environment modify the relationship between intervention and fruit and vegetable serving change among low-income populations. The main hypothesis being tested is that the intervention effects on fruit and vegetable serving changes will be greater among intervention participants relative to control participants for the following food environment measures: (1) neighborhood availability of food stores (objective and perceived); (2) in-store availability of healthy foods (objective and perceived); (3) store accessibility (objective and perceived).

## 2. Methods

### 2.1. Weightwise Intervention

#### 2.1.1. Study Sample

**Participant Inclusion/Exclusion Criteria**

Women between 40–64 years who met the following inclusion criteria were eligible for study participation: (1) BMI between 27.5 and 45 kg/m^2^, inclusive; (2) willing to lose 5% or more of initial body weight and follow recommendations for healthy dietary and physical activity patterns; (3) English speaking; (4) able and willing to give informed consent; (5) household income less than or equal to 250% of federal poverty guidelines. The following exclusion criteria were also applied: (1) medical or physical limitations to engaging in moderate-intensity physical activity; (2) medical or other contraindications to weight loss; (3) history of gastric bypass surgery or scheduled surgery for this purpose; (4) weight loss of >20 pounds in the last 3 months; (5) current use of medication for weight loss; (6) treatment of psychosis; (7) diagnosis of type 1 diabetes. Finally, women who were pregnant, breastfeeding, or planned to get pregnant or relocate (more than 50 miles from their health department) in the next year and a half were ineligible.

**Recruitment**

Women were recruited from 6 county health departments in North Carolina (Davidson, Forsyth, Lincoln, Warren, Nash, and Pasquotank) representing 3 counties in the eastern part and 3 counties in the western part of North Carolina. Four of the six counties are classified as nonmetro; two are classified as metro [16] based on rural-urban continuum codes. A total of (*n* = 162) women were enrolled in the intervention study. Complete details of the study design, intervention components, and baseline characteristics have been published elsewhere [17, 18]. The University of North Carolina School of Medicine Institutional Review Board approved and monitored this study.

#### 2.1.2. Study Design

A randomized controlled trial study design conducted in 2008-2009 was used to test the effectiveness of a 16-week evidence-based behavioral weight management intervention (the Weightwise program). The intervention design and baseline results have been described elsewhere [19]. Briefly, eligible participants were measured at baseline and then randomized to either delayed intervention (DI) group or the special intervention (SI) group, which received the 16-week intervention. The DI group participants were controls, receiving no treatment, for the 16-week intervention time period. The DI participants received the intervention after the control trial study period ended. The weight loss goal for the study is 10 or more pounds (≥4.5 kg).

#### 2.1.3. Weightwise Intervention Components

The components of the intervention are based on previously developed and tested intervention materials. Diabetes prevention program leader's guides [20] and adaptations of the PREMIER [21] guides for the weight loss maintenance (WLM) trial were further adapted for low-income midlife women [17].

Weekly group sessions included 1.5 to 2 hours of face-to-face contacts delivered in the following format: “check-in”, “session topic” (e.g., stress, eating out, healthy eating on a budget, how to stretch your food dollar, social and environmental factors that help with diet change, or portion sizes), “activity” related to diet or physical activity (e.g., cooking skills, meal preparation, and recipe tasting), and goal-setting and action planning.

### 2.2. Food Environment Measures

#### 2.2.1. Self-Report of Food Shopping Habits

Participants were asked where they conduct their primary food shopping as well as other stores where they purchase food at baseline and after intervention. Participants were also asked about the amount they spend on groceries per week and how often they food-shop at baseline and after intervention.

#### 2.2.2. Measurement of Participants' Food Environment

The food environment of participants from the total sample of the Weightwise study was characterized in three ways during the intervention period of 2008-2009: (1) the number and type of food stores within the census tract; (2) availability of healthy foods in stores where participants shop, as measured by food store audits; (3) an aggregate score of self-reported access to healthy foods in the primary food store, availability and affordability of healthy foods in their neighborhood and primary food stores (perceived measures).

#### 2.2.3. Objective Measure of Neighborhood Store Availability

To create the objective neighborhood store variable, three steps were conducted. *Step (1)*. Data on the number and type of food stores in all 6 counties were obtained from InfoUSA, Inc. (Papillion, Nebraska) in August 2008 and 2009 to assure accuracy in address over repeated times. Food stores were classified based on supplemented Standard Industrial Classification codes (SIC codes) as used by previous studies [22, 23], across the following categories: supercenters, convenience stores, and supermarkets and large chain grocery stores combined. *Step (2)*. we mapped the number and type of stores in each participant's census tract. To conduct this step, we gathered each participant's home address, geocoded and matched their address to the 2000 US census tract using Juice analytics software (http://www.juiceanalytics.com) and ArcMap (ArcGIS version 9.2, 1999–2994; ESRI, Redlands, CA, USA). *Step (3)*. Objective neighborhood availability was dichotomized as either “yes” (≥1) for each store type in census tract or “no” (none of that store type) based on previous literature [23].

#### 2.2.4. Objective Measure of Food Availability in Primary Food Store

Specific food stores where women shopped were identified from a survey question asking, “What is the name and street of the grocery store where you do your primary shopping?” Women were also asked, “What is the name and street of a second store where you conduct food shopping?” The survey responses regarding the location of the named food store were confirmed using groundtruthing (direct observation of food store addresses) (1,34). The stores where women reported shopping corresponded to their census tract. However, a large proportion of the women reported shopping at a second store not in their census tract (43%). For the purposes of this study, we only conducted food store assessments in the primary food store. To ascertain in-store food availability, we modified items from the Nutrition Environment Measures Survey in Stores (NEMS-S) (35) using data about purchasing habits from the Bureau of Labor Statistics (36) and the US Department of Agriculture Continuing Survey of Food Intakes by Individuals (CSFII). To reflect the purchasing habits of the Weightwise study population (low-income southern women), we therefore added frozen and canned goods and pork, while excluding baked goods (37). In the spring and summer of 2009 (after participants had been enrolled into the study), we assessed food availability in all eighty stores identified by participants, focusing on thirty-seven food items in nine food groups: (i) nonfat/low-fat milk; (ii) fruit; (iii) vegetables; (iv) low-fat meats; (v) frozen fruit and vegetables; (vi) canned vegetables; (vii) 100% whole-wheat bread; (ix) non-sugar-sweetened cereals. All stores were surveyed between 09.00 and 16.00 hours on weekdays to maintain consistency relative to stock on the shelves between stores. The audits were conducted among a graduate student and author trained in the NEMS-S protocol (http://www.med.upenn.edu/nems/measures.shtml). A tally sheet was used to determine whether the food item was available at the time of the audit. Each food item received 1 point if available, with a minimum possible survey score of zero and a maximum possible score of 37. Food store availability then was categorized as low, medium, or high (tertiles) to facilitate comparisons with other studies (38) and based on distribution of the data.

#### 2.2.5. Measure of Perceived Healthy Food Availability, Accessibility, and Affordability in Neighborhoods and Primary Food Stores

Participants' baseline perception of their local food environment was collected via a phone survey after enrollment into Weighwise but before the intervention began. The survey was then administered again among all participants after intervention. The survey questions were used to measure perceived access and availability of healthy foods in each person's neighborhood (defined as the area approximately 5 miles around her home with correspondence to the size of the census tract), as well as availability, and affordability of healthy foods in the primary food store. Each measure is described in detail below.

#### 2.2.6. Neighborhood Healthy Food Availability

To assess perceived neighborhood healthy food availability, participants were asked about the extent to which they agreed with the following statements about their neighborhood: (i) “A large selection of fruits and vegetables is available in my neighborhood”; (ii) “A large selection of low-fat products is available in my neighborhood”; (iii) “The fruits and vegetables in my neighborhood are of a high quality.” Responses to all questions were coded on a 5-point Likert scale (0 = “strongly agree”; 4 = “strongly disagree”). There are 3 questions each worth a possible 4 points, for a total of 12 points (4∗3). The neighborhood availability questions have previously been tested for reliability and validity and are described elsewhere (4,40).

#### 2.2.7. In-Store Healthy Food Availability and Affordability

Participants were then asked about the extent to which they agreed with the following statements for their primary food store: (1) “A large selection of fruits and vegetables is available”; (2) “A large selection of low-fat meat products is available (90% lean beef, skinless chicken)”; (3) “A large selection of brown breads is available”; (4) “A large selection of low-fat cheese or skim milk is available.” The above 4 questions were asked to assess affordability. The in-store availability had 4 questions each worth possible 4 points, for a total possible score of 16 (4∗4). The in-store affordability had 4 questions each worth possible 4 points, for a total possible score of 16 (4∗4). The range of scores on these measures was 0 to 16 with a higher score indicating higher perceived availability or affordability.

Each of the above questions was used to create a summary measure. The three summary measures used in analyses were (1) neighborhood availability; (2) food store availability; (3) food store affordability. These three variables and the corresponding scoring procedures are based on the reliable and valid results of previous studies [24]. The three measures were categorized into high, medium, and low availability or affordability (tertiles) based on nonnormal distribution of data.

#### 2.2.8. Accessibility

Access was defined in two ways: (1) objective potential spatial access which was calculated from the network distance from the participant's home to their primary food store; (2) perceived access by asking about length of time and distance traveled to the primary food store.

A dichotomous variable was created to group access into easy or difficult access. Easy access was defined as living less than 5 miles to the primary food store as first inclusion criteria, followed by living less than 10 minutes. Difficult access was defined as living 5 miles or greater, followed by living greater than 10 minutes. The cut points of 5 miles or 10 minutes correspond approximately to the mean response (average distance traveled to primary store was 4.7 miles Table 1) and are also consistent with the cut points used in prior studies [25].

### 2.3. Definition of Outcomes

#### 2.3.1. Fruit and Vegetable Intake

Fruit and vegetable intake was collected using a validated rapid food frequency survey [26], which assessed fruit, vegetable, and fiber intake. The survey is effective in identifying persons with high fat, low fruit/vegetable intake, or low fiber intake. The fruit and vegetable serving change outcome was calculated from the Block score. The Block score is derived from calculating nutrient intakes based on answers to questions on frequent consumption of fruits and vegetables. Change in fruit and vegetable serving was calculated as the difference between baseline and postintervention scores.

### 2.4. Statistical Analyses

One hundred sixty-two (162) women were enrolled and randomized into either the DI or SI group. Of the 162 participants, 4 were missing data on fruit and vegetable intake, and 2 were missing data on the perceived food environment survey. The final analytic sample consisted of 156 women with complete data on all exposure and outcome variables. The 6 women dropped from analyses were not significantly different from the final sample based on key demographics (race, age, and fruit and vegetable intake).

Data analyses included descriptive statistics, *t*-tests, and Pearson chi-square to compare baseline and post intervention perceived food environment survey results within intervention and control participants. Multivariable general linear regression analysis was used to model the association between the intervention effect of fruit and vegetable serving change and each food store environment measure as a modifier in that relationship; the intervention effect on fruit and vegetable serving change will be modified by each food environment variable. For each model, an interaction term was used between fruit and vegetable serving change and the food environment variable (f/v_serving ∗ perceived food environment). If the term was significant at *P* < 0.05, it was included in the model for the intervention effect on fruit and vegetable serving change. All models included a cluster statement on county since women are nested within the 6 counties, allowing for calculation of robust standard errors. Huber-White sandwich variance calculates the variance allowing for nonindependence. The type I error rate was set at 0.05 for main effects and interaction terms for all models. 95% confidence intervals were calculated for all models. All analyses were conducted in Stata 11.0 [21].

## 3. Results

Table 1 shows the baseline demographics and perceived and objective food environment scores for intervention and control participants. There were no statistically significant differences between control and intervention participants. The mean BMI was 37.5 (±4.7 both intervention and control) with an initial weight of 220 (±35 intervention and ±28 control) pounds in both groups. Both groups consumed about 4 servings of fruits and vegetables per day. On average, both groups perceived their neighborhood above average in availability of healthy foods (8.5 ± 3.0 intervention and 8.2 ± 3.1 control out of 12 points) and their primary food store as having above average in availability of healthy foods (12.7 ± 2.3 intervention and 12.6 ± 2.6 control out of 16 points). Overall, participants reported that their primary grocery store had many fruits, vegetables, and low-fat foods. The mean number of supercenters was slightly higher among controls (0.23 ± 0.42) relative to intervention participants (0.17 ± 0.38). Additionally, both groups lived in neighborhoods with similar mean number of supermarkets (0.83 ± 0.38 intervention and 0.85 ± 0.36 control) and mean number of convenience stores (0.89 ± 0.32 intervention and 0.85 ± 0.36 control).

Table 2 shows the changes in perceived food environment measures among intervention and control participants. Significant changes between baseline and postintervention were found among intervention participants for the following: an increase in the amount of money spent on groceries and an increase in the score for perception of affordability of food in their primary grocery store. Interestingly, significant increases between baseline and postintervention were found among control participants for those shopping at road side stands and homegrown gardens. Lastly, there was an increase in perception of in-store availability of healthy foods among controls.

Table 3 shows the results from determining if the food store environment modifies the intervention effect on fruit and vegetable serving change. Of all perceived and objective measures tested for interaction, the following three models produced significant results (*P* < 0.05): (1) perceived in-store availability; (2) perceived in-store availability of low-fat foods; (3) objective neighborhood store availability. Among intervention participants who perceived their store low in availability of healthy foods at baseline, they had an increase of almost 2 servings of fruits and vegetables [1.89, (95% CI 0.48, 3.31)] compared to controls that perceived their store low in availability. Additionally, intervention participants who perceived their store low in availability of low-fat foods at baseline had an increase of almost 2 servings of fruits and vegetables [1.85 (95% CI 0.87, 2.82)] compared to controls that perceived their store low in availability of low-fat foods. Lastly, intervention participants that lived in a neighborhood with a low store density of supermarkets had a greater intervention effect on fruit and vegetable serving change [1.62 (95% CI 1.27, 1.96)] relative to controls with a low store density of supermarkets.

## 4. Discussion

The study finding of perceived and objective measures modifying the relationship between an individual-level intervention with diet change offers insight into the complex relationship between the individual and their food store environment.

A unique contribution to the field is this study's capture of perception within a behavioral intervention. Results in changes of perception of the food environment among intervention participants suggest that although the mean amount spent on groceries increased, the perception of their food store having affordable healthy items increased as well. The increase in perceived affordability within their store suggests that the intervention may have indirectly brought about awareness about healthy food options that are affordable where women shop. The finding suggests that an intervention at the individual level can influence perception at the environmental level. A surprising finding was the change among controls between baseline and postintervention in increasing the number of times shopping at road side stands or homegrown gardens. Cogent to this was the increased perception of healthy food being available in their neighborhoods. These findings go hand in hand given the recent evidence linking community gardens with increased consumption of fruits and vegetable servings and community connection [10, 27]. Although the control participants did not receive any intervention, they were aware that they would be participating in a weight loss intervention in the near future. The anticipated participation perhaps led to a heightened awareness of the food available for purchase in their neighborhood. Through this awareness, they were more likely to seek food items that were close by and affordable, such as road side stands and homegrown gardens.

A distinctive approach to our study was measuring the interaction between perception of the food environment with fruit and vegetable serving change. Results highlighting the complex relationship between what people subjectively think of their environment and how that might modify the success of the intervention are reflected in the result of fruit and vegetable serving change among women who perceived their store as not having many healthy food items. Among women in the intervention group, those who perceived their store low in availability had a greater increase in consumption of fruits and vegetable servings, compared to control participants with a low perception of their store. A possible explanation for this association is that the intervention brought about an increased awareness in the availability, or the lack thereof, of fruits and vegetables in the store where women shop. Women who perceived their store low in availability at baseline then learned where to locate affordable fruits and vegetables through participation in the intervention. The intervention sessions discussing affordable healthy options helped to increase knowledge and awareness about where to buy fruits and vegetables in their community. This heightened awareness was transferred into action by augmenting their shopping habits and therefore consuming more fruits and vegetables.

The intervention effect on fruit and vegetable change varies by neighborhood density of supermarkets. Among women in the intervention group, those who live in neighborhoods with no supermarkets, these women had a greater increase in fruit and vegetable serving intake compared to controls without a supermarket in their neighborhood. This result points to the possible individual ability to overcome a neighborhood with poor availability and still improve fruit and vegetable servings through an effective intervention. Given that a recent study has found no association with proximity to supermarkets and fruit and vegetable intake [28], perhaps proximity to supermarkets is not as relevant as having the skills and efficacy to make healthy choices. Additionally, many women reported shopping at nontraditional food stores as a secondary store, Table 2. The intervention might have brought to attention the ability for low-cost healthy options at these non-traditional food outlets, which helped to increase fruit and vegetable servings.

Our result not finding an association between high density of supermarkets with improved diet is not surprising given a recent study finding no associations between neighborhoods with supermarkets and diet [28]. Additionally, based on previous results [3], it appears as though in rural landscapes that neighborhoods with supermarkets may represent areas and stores with high availability of energy dense foods [29, 30] which overrides any benefit of availability of healthy food options [31]. This high availability of energy dense foods leads to less intake of fruits and vegetables regardless of the intervention effect.

There are several limitations to this study. First, the small sample size limits the power and ability to detect associations. Second, objective food store addresses were collected from secondary data sources. The data source may misrepresent the true food store environment [32, 33]. Additionally, store classification was confined to supercenters, supermarkets, and convenience stores. As results suggest, a substantial percentage of women also shop at nontraditional food outlets such as Dollar Stores, which were not captured in defining the food neighborhood. The inclusion of such stores may add information about the true food store environment not depicted in this study. Third, the use of census tract for defining neighborhood may not reflect individual's true neighborhood habits and exposure level. It is likely that some of the women living in the residential census tract where objective food store exposure was assigned do not actually define that area as their neighborhood. However, this misclassification of exposure is not likely to be associated with the outcome of diet or weight such that women who define their neighborhood in a different spatial area are not more or less likely to be overweight or have a poor diet, and therefore unlikely to have biased our estimates. Fourth, multiple models were tested, and results may be due to chance alone. Therefore, results need to be confirmed in future studies. However, given the study design of a randomized trial along with the in-depth data collection, the results found suggest the food environment may modify dietary outcomes within an intervention.

## 5. Conclusion

This study contributes to a growing body of research examining how the food store environment is associated with diet and weight, especially among low-income populations. Results point to how the food store environment may modify the success of a behavioral weight loss intervention on diet change among low-income women. Interventions may need to incorporate strategies aimed at where an individual lives and shops to help improve dietary outcomes.

## Figures and Tables

**Table 1:** Baseline participant characteristics mean (SD) or percent, North Carolina, 2009.

	Intervention	Control
	*n* = 92	*n* = 64
*Demographics*		
Age (years)	52 (7.4)	52 (6.7)
Education (years)	13 (1.9)	13 (1.8)
Smoke (%)	14%	17%
Employed full time (%)	35%	23%
Income (≤$29,000)	67%	68%

*Race*		
White	40%	42%
African American	59%	55%
Other	1%	3%

*Baseline Weight and Diet*		
BMI kg/m²	37.5 (4.7)	37.4 (4.7)
Weight lbs	220 (35)	220 (28)
Fruit and vegetable servings	4 (1.8)	4 (1.4)

Perceived healthy food in neighborhood*		
Availability (range 0–12)	8.5 (3.0)	8.2 (3.1)

Perceived healthy food in primary food store*		
Availability (range 0–16)	12.7 (2.3)	12.6 (2.4)

Perceived healthy food affordability in primary food store*		
Affordability (range 0–16)	9.6 (3.4)	9.5 (3.4)

Perceived primary food store access**		
Miles	4.7 (5.0)	5.7 (5.9)

Objective neighborhood availability*		
Supercenters	0.17 (0.38)	0.23 (0.42)
Supermarkets	0.83 (0.38)	0.85 (0.36)
Convenience	0.89 (0.32)	0.85 (0.36)

Objective food store availability*		
Food store score	34.7 (2.5)	33.8 (4.4)

Objective access**		
Miles	6.0 (6.2)	5.6 (5.6)

*Higher scores indicate greater availability or affordability of healthy foods at the store and neighborhood level.

**Access = reported or calculated miles from home to primary food store.

**Table 2:** Pre- and Postintervention perceptions of the food store environment (mean (SD) or percent), North Carolina, 2009.

	Intervention (*n* = 92)			Control (*n* = 64)		
	Before	After	Change	Before	After	Change
*Primary Store*			*P* = 0.24			*P* = 0.78
Supermarket	71%	63%		79%	79%	
Supercenter	29%	37%		21%	21%	

*Frequency of shopping*			*P* = 0.14			*P* = 0.78
2 or more times per week	46%	43%		45%	53%	
1 time per week	25%	30%		21%	25%	
2 to 3 times per month	22%	22%		30%	19%	
1 time per month	9%	4%		4%	4%	

*Amount spent on groceries (dollar)*	196 (129)	238 (153)	*P* = 0.04	189 (126)	195 (120)	*P* = 0.79

*Second store for food shopping*						
Dollar store	39%	33%	*P* = 0.30	49%	36%	*P* = 0.45
Grocery store	49%	53%	*P* = 0.21	38%	60%	*P* = 0.23
Supercenter	54%	42%	*P* = 0.24	60%	58%	*P* = 0.19
Farmer's markets	20%	13%	*P* = 0.20	19%	13%	*P* = 0.74
Road side stand	8%	8%	*P* = 0.43	4%	9%	*P* = 0.05
Homegrown garden	17%	22%	*P* = 0.73	11%	23%	*P* = 0.01

*Perceived neighborhood*	8.5 (3.0)	9.0 (2.5)	*P* = 0.25	8.2 (3.1)	9.2 (2.6)	*P* = 0.11

*Perceived in-store availability*	12.7 (2.3)	13.2 (2.5)	*P* = 0.37	12.6 (2.4)	13.2 (2.5)	*P* = 0.03

*Perceived affordability*	9.6 (3.4)	10.6 (3.4)	*P* = 0.004	9.5 (3.4)	11 (3.6)	*P* = 0.34

*P* ≤ 0.05						

**Table 3:** Intervention effect on fruit and vegetable serving change within each level of food store environment measures, North Carolina, 2009.

	Fruit and vegetable serving change (95% confidence intervals)
*Perceived*	
*In-store availability (a)*	
Low availability	1.89 [0.48, 3.31] *P* = 0.04
Medium availability	0.33 [−0.92, 1.58]
High availability	−0.66 [−3.14, 1.82]
*In-store low-fat (b)*	
Low-fat availability (low)	1.85 [0.87, 2.82] *P* = 0.03
Low-fat availability (high)	0.01 [−1.00, 1.02]

*Objective *	
*Neighborhood store availability*	
*Census tract (c)*	
Supermarket (low density)	1.62 [1.27, 1.96] *P* = 0.03
Supermarket (high density)	0.05 [−1.02, 1.11]

(a)–(c) Reference for each model is control group for that level of perceived or objective measure.

*P* ≤ 0.05.
